# Two-dimensional ETTC–labeled longitudinal and lateral conflicts for interpretable real-time crash risk prediction in freeway interchange diverging areas

**DOI:** 10.1371/journal.pone.0344623

**Published:** 2026-04-29

**Authors:** Feng Tang, Rui Wu, Zhizhen Liu, Shiyu Zhong

**Affiliations:** 1 Engineering Research Center of Catastrophic Prophylaxis and Treatment of Road & Traffic Safety of Ministry of Education, Changsha University of Science & Technology, Changsha, Hunan, China; 2 School of Transportation, Changsha University of Science & Technology, Changsha, China; 3 Guangzhou Baiyun International Airport Co., Ltd., Guangzhou, China; Southwest Jiaotong University, CHINA

## Abstract

Current real-time crash prediction models (RTCPMs) for freeway diverging areas primarily rely on macroscopic traffic parameters, which inadequately capture how vehicle interactions escalate into crash risks. This study analyzed 12 interchange diverging areas from two multilane freeways in China, employing image recognition technology to extract 48 vehicle motion parameters and surrogate safety measures (SSMs). Extended Time-to-Collision (ETTC)—a validated two-dimensional metric for lateral conflicts—was innovatively applied to establish a refined database with longitudinal/lateral conflict labels at 30-second intervals. Following spatiotemporal conflict analysis, four RTCPM types—Random Forest, Neural Network, Support Vector Machine, and XGBoost—were developed, with SHAP interpretability framework analyzing key risk factor contributions. Results showed: 1) XGBoost achieved optimal performance; 2) lateral conflicts exhibited longer durations and higher crash risks than longitudinal conflicts, with severe conflicts concentrated within 200 meters upstream of exit ramps; 3) SSMs including Modified Time-to-Collision (MTTC)—which incorporates relative acceleration—alongside Stopping Headway Distance and Time-to-Collision, emerged as decisive factors for both crash types, ranking highest in predictive contribution. These findings provide scientific foundations for designing dangerous driving warning systems and implementing proactive traffic safety management at interchange diverging areas.

## 1 Introduction

Freeway interchange diverging areas witness a high frequency of traffic accidents due to recurrent lane-changing maneuvers. These areas account for 37.2% of all interchange crashes and have a fatality rate 42% higher than that of typical road segments [1]. Active traffic safety management technologies monitor real-time conditions, detect potential hazards, and execute immediate interventions. Consequently, they effectively mitigate traffic accidents and represent a mainstream strategy in contemporary freeway safety management [2].

The Real-time Crash Prediction Models (RTCPMs) are critical components of active traffic safety management systems, referring to methods that utilize real-time traffic flow parameters to predict whether crash events will occur within a short time window. However, although crash records remain a widely used data source for real-time crash prediction models (RTCPMs), they are often sparse and subject to reporting delays and timing inaccuracies. With advances in high-resolution sensing technologies and trajectory data availability, an increasing number of studies have utilized traffic conflicts quantified by surrogate safety measures (SSMs) as proactive proxies for crash risk. These measures provide richer pre-crash behavioral information and help alleviate the data sparsity inherent in crash-only modeling approaches [3–6]. Therefore, a crucial aspect of the RTCPM research framework is the selection of appropriate surrogate safety measures (SMMs).

Due to limitations in data collection technologies, many existing studies continue to rely on crash records reported by traffic police, which may deviate from actual crash timings and are often sparse, thereby compromising model performance. To address these limitations, traffic-conflict-based surrogate safety measures (SSMs) have been extensively investigated. Recent studies have systematically compared different SSM candidates and provided purpose-oriented indicators and selection guidance for various facilities and applications [7,8]. Moreover, unlike other freeway segments, the diverging areas of interchanges involve frequent lane-changing due to diverging tasks, resulting in fundamentally different mechanisms for longitudinal and lateral conflicts, which necessitate differentiated treatment.

**Fig 1:**
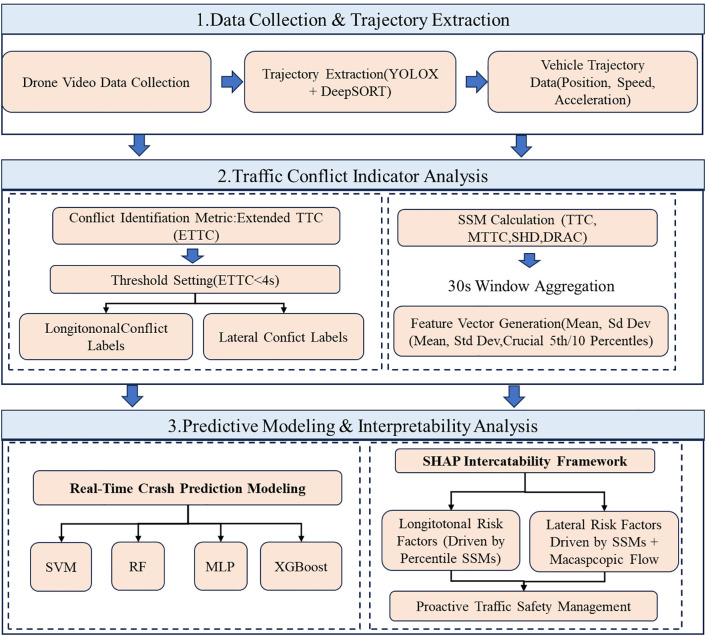
Technical roadmap of this study.

Building on the established body of conflict-based real-time crash prediction modeling (RTCPM) research, this study advances proactive safety management in freeway interchange diverging areas by developing a conflict-type-aware and interpretable real-time crash risk prediction framework. The key novelty lies in explicitly operationalizing and modeling lateral lane-changing risk, rather than implicitly mixing it with longitudinal car-following risk. Specifically, this study (1) constructs a high-resolution trajectory-based dataset covering 12 freeway diverging areas and extracts microscopic motion descriptors and surrogate safety measures (SSMs); (2) introduces a 30-s traffic-state labeling scheme that distinguishes longitudinal and lateral conflicts, in which lateral interactions are identified using the two-dimensional Extended Time-to-Collision (ETTC) to accommodate multi-angle vehicle interactions in diverging zones; (3) develops and benchmarks multiple machine-learning-based RTCPMs (RF, MLP, SVM, and XGBoost) for conflict-type-specific risk prediction; and (4) integrates SHAP-based interpretability analysis to identify mechanism-consistent key risk drivers and translate them into spatially and temporally targeted proactive traffic control implications (e.g., where and when longitudinal versus lateral risks dominate). The technical roadmap of the proposed framework is illustrated in Fig 1.

The main contributions are threefold: (i) an ETTC-enabled lateral-conflict identification and 30-s conflict-type labeling pipeline for diverging areas; (ii) a comparative evaluation of conflict-type RTCPMs on multi-site diverging-area trajectory data; and (iii) an interpretable risk-factor diagnosis that supports proactive control strategies.

## 2 Literature review

Based on the typical RTCPM research framework, this paper primarily reviews the data collection methods and types, the definitions of pre-collision traffic conditions, and the research progress and trends of safety surrogate measures.

### 2.1 Data collection methods and types

Since loop detectors have been widely installed and used for traffic management in many countries, they have held a dominant advantage in traffic data collection over the past two decades [9–14]. The types of information typically collected include traffic volume, occupancy time, and speed [9,12,13]. With the advancement of roadside sensing technologies—especially the application of fine-grained sensing methods such as video, microwave radar, and LiDAR—more detailed descriptions for traffic data detection and analysis have become available. These include indicators such as Time Headway (THW), Time-to-Collision (TTC), Post-Encroachment Time (PET), and both longitudinal and lateral acceleration [14,15]. These fine-grained sensing data not only provide a better description of risks, but are also considered to offer improved capabilities in risk prediction [16]. In terms of data types, the mean and standard deviation of traffic flow, occupancy, and speed are the most commonly used variables, appearing in nearly all RTCPM modeling studies [17–20]. However, Wang et al. [21] proposed using the mean deviation and standard deviation of time headway and space headway to describe car-following behavior for improved prediction performance. Basso et al. [22], based on the spectrum of car-following behavior, selected three indicators—reciprocal of time-to-collision, lateral swing coefficient, and speed instability coefficient—to characterize car-following risk states. Additional environmental variables, such as visibility [23], weather conditions [24], road conditions [25] and vehicle type [26], have also been taken into account.

### 2.2 Definition of pre-crash traffic conditions

Pre-crash traffic conditions generally refer to the state of traffic flow prior to a collision. A common method for defining the pre-crash period is to segment time into 5-minute intervals and then aggregate and analyze the traffic flow data within each interval [16,-28–34]. For example, Zhao et al. [27] modeled the six 5-minute intervals before a crash and found that models based on intervals closer to the time of collision yielded better predictive performance. Zheng et al. [16] developed a real-time crash risk model for highways using three types of basic traffic flow data from detectors located upstream and downstream of the crash site during the 10–40 minutes preceding the incident, achieving high prediction accuracy. Gu et al. [28] used support vector machines to build real-time rear-end crash prediction models based on traffic data from 5–10 minutes, 10–15 minutes, and 15–20 minutes before the crash. Their results showed that the model based on data from 5–10 minutes prior was most effective for real-time prediction. In addition, other scholars have explored the influence of finer-grained traffic data on highway crash risk. For instance, Wang et al. [29] extracted real-time traffic data collected at 30-second intervals, along with crash records, to identify hazardous traffic flow conditions.

### 2.3 Surrogate safety measures

#### 2.3.1 Surrogate safety measures.

Surrogate Safety Measures (SSMs) are quantitative indicators that assess road traffic safety without relying on actual crash data, instead evaluating risk based on traffic conflicts, vehicle operating states, or potential hazardous scenarios [30–34]. Since their emergence in the late 20th century, SSMs have become fundamental tools in proactive traffic safety research, addressing the inherent limitations of crash-based analyses including data scarcity, underreporting, and the inability to capture pre-crash behavioral dynamics [35].

#### 2.3.2 Classification and evolution of SSMs.

Surrogate safety measures (SSMs) can be computed at different observational levels. Some indicators are derived from the state of a single vehicle (e.g., speed- or acceleration-based proxies reflecting vehicle control instability) [32,35]. In contrast, conflict-oriented SSMs are fundamentally interaction-based and are derived from the spatiotemporal relationship between a subject vehicle and a counterpart, typically a leader/follower or an adjacent vehicle [33]. In this study, the term SSMs is used specifically to denote interaction-based surrogates that quantify proximity, required evasive effort, or safety margins between two vehicles, whereas single-vehicle kinematic statistics are treated as vehicle motion parameters that complement SSMs by characterizing the underlying driving dynamics that give rise to vehicle interactions.

Surrogate safety measures (SSMs) are commonly classified into three categories: time-based, deceleration-based, and distance-based measures. Time-based indicators (e.g., TTC, MTTC, PET, and THW) characterize temporal proximity and are widely adopted due to their intuitive interpretation and computational efficiency [35,36]. However, classical TTC relies on restrictive assumptions (e.g., constant speed and linear motion) and may underestimate crash risk under transient disturbances or nonlinear dynamics [31]. To address these limitations, recent studies have proposed dynamics- and severity-aware extensions, such as Time-Integrated TTC (TIT), which accumulates both the magnitude and duration of unsafe TTC states [37]. Another representative extension is Time to Collision with Disturbance (TTCD), which accounts for abrupt lead-vehicle deceleration to better represent rear-end crash risk under traffic perturbations [37,38]. For lateral interactions, extended TTC formulations have also been applied to capture two-dimensional proximity during lane-changing maneuvers.

Deceleration-based SSMs (e.g., DRAC and required deceleration measures) focus on the braking effort required to avoid a collision and therefore provide a stronger connection to vehicle control limits [37]. However, their performance can be sensitive to threshold selection and may neglect heterogeneity in driver response times and vehicle capabilities. Recent improvements include Modified DRAC (MDRAC), which incorporates response-time effects, as well as probabilistic constructs such as the Crash Potential Index (CPI), which estimate the likelihood that the required deceleration exceeds feasible limits [36,39]. Compared with deterministic cutoffs, probabilistic formulations enhance interpretability and robustness when traffic participants exhibit diverse control behaviors.

Distance-based measures (e.g., SHD and PSD) reflect spatial safety margins and are useful for identifying margin depletion; however, when used alone, they often cannot distinguish stable close-following from rapidly deteriorating interactions [40]. Consequently, recent studies increasingly advocate combining distance-based indicators with time- and deceleration-based measures to obtain a more comprehensive description of traffic conflict mechanisms [32,39].

Beyond single metrics, recent studies have emphasized hybrid and composite SSMs that integrate multiple dimensions of risk, as exemplified by kinematics-informed measures such as PICUD [40]. This trend reflects an emerging consensus that traffic conflicts are inherently multi-attribute phenomena and cannot be reliably characterized using a single indicator.

A major research frontier concerns validation, threshold transferability, and the treatment of uncertainty. Although statistical approaches, such as extreme value theory and Bayesian hierarchical modeling, have strengthened empirical conflict–crash linkages, fixed thresholds remain difficult to generalize across varying contexts, particularly in mixed traffic environments involving heterogeneous human-driven and automated vehicles. Recent review studies highlight the lack of a unified framework for selecting, calibrating, and fusing SSMs and thresholds, thereby motivating adaptive, probabilistic, and uncertainty-aware approaches [41,42]. Overall, the evolution of SSMs suggests that future progress will depend less on proposing isolated new measures and more on developing integrated and transferable frameworks that support multi-metric fusion and real-time safety inference.

#### 2.3.3 Contemporary applications and emerging indicators.

The advent of connected and automated vehicle (CAV) technologies has catalyzed significant innovation in SSM development. Traditional measures designed for human-driven vehicles exhibit limitations when applied to mixed traffic flows with varying automation levels. Wang et al. [43] recently proposed Time to Avoid a Crash (TTAC), a novel indicator specifically calibrated for emerging mixed traffic scenarios at signalized intersections. Through simulation experiments across CAV Market Penetration Rates (MPRs) ranging from 0% to 100%, TTAC demonstrated superior sensitivity in identifying conflicts involving automated vehicles compared to conventional TTC and PET measures. This advancement underscores the necessity for context-specific SSM adaptation as traffic composition evolves.

Data acquisition methodologies for SSMs have similarly progressed from manual observation to automated extraction from two primary sources: (1) onboard sensor data capturing ego-vehicle surroundings and motion parameters through radar, LiDAR, and GPS systems; and (2) roadside sensing data recording vehicle trajectories within road segments via fixed cameras and detectors [44]. High-resolution trajectory datasets—such as those from the Next Generation Simulation (NGSIM) program and naturalistic driving studies—have enabled microscopic analysis of conflict precursors, facilitating machine learning-based real-time risk prediction [22].

#### 2.3.4 Contemporary applications and emerging indicators.

Despite extensive SSM literature, three critical gaps persist: (1) limited application to terrain-influenced scenarios where road gradients alter braking dynamics; (2) insufficient integration of spatiotemporal heterogeneity in conflict risk across interchange geometric configurations; and (3) lack of interpretable machine learning frameworks linking multiple SSMs to crash likelihood in real-time operational contexts.

For this study, we selected five complementary SSMs to address these gaps:

- TTC and MTTC: Capture temporal urgency while MTTC incorporates relative acceleration, improving sensitivity to dynamic traffic states [36].
- SHD: Explicitly accounts for road gradient effects on stopping distances—critical for mountainous freeway interchanges where grades exceed ±3%.
- DRAC: Quantifies kinematic severity of required evasive actions.
- PICUD: Provides hybrid temporal-kinematic assessment validated for freeway weaving/merging conflicts.

This multi-dimensional SSM suite enables comprehensive conflict characterization across the temporal (when), spatial (where), and severity (how critical) dimensions essential for interchange diverging area safety analysis. The selection prioritizes indicators with: (1) established theoretical validity, (2) proven sensitivity to freeway-specific conflict types, and (3) computational feasibility for real-time prediction frameworks—criteria aligned with contemporary best practices in SSM-based safety modeling [44,22].

### 2.4 Summary of current research status and research objectives

RTCPMs and their key influencing factors on freeways has several limitations. First, limited by detection technologies, the feature variables used in previous RTCPMs primarily relied on macroscopic traffic flow parameters from fixed-point detectors. Consequently, microscopic surrogate safety measures (SSMs)—which are strongly associated with crash risk—were not incorporated. This omission severely compromises RTCPM performance. Second, although studies have identified key factors affecting crash risk on basic freeway segments and urban expressways, research focusing on interchange diverging areas in mountainous freeways remains scarce. As critical nodes, interchange diverging areas experience more complex traffic conditions than other segments. Notably, frequent lane-changing maneuvers further complicate the mechanisms governing crash risk.

This study aims to develop a Real-Time Crash Prediction Model (RTCPM) capable of distinguishing between different types of traffic conflicts (longitudinal vs. lateral) in freeway interchange diverging areas, and to identify the key risk factors driving each conflict type. To achieve these objectives, a machine learning-based framework is proposed with three core components:

(1) Surrogate Safety Measure (SSM) Extraction: Vehicle trajectory data from drone videos are processed to compute 48 motion parameters and SSMs (e.g., ETTC, MTTC, TTC, SHD, DRAC). These microscopic indicators capture vehicle interaction dynamics that macroscopic traffic flow parameters cannot reveal.
(2) Model Development and Comparison: Four candidate algorithms—XGBoost, Random Forest (RF), Neural Network (MLP), and Support Vector Machine (SVM)—are trained and evaluated to determine the most effective approach for this application. The selection criteria prioritize accuracy, recall (to minimize missed conflicts), and low false positive rates (to avoid alarm fatigue).
(3) Interpretability Analysis: The SHAP (SHapley Additive exPlanations) framework is applied to the best-performing model (XGBoost) to quantify the contribution of each feature to predictions. This analysis identifies which traffic parameters most strongly influence longitudinal and lateral crash risks, providing a scientific basis for targeted safety interventions.

Through this framework, machine learning serves not merely as a “black-box” predictor but as an interpretable tool that bridges microscopic vehicle behavior and segment-level crash risk, directly addressing the research gap.

## 3 Data collection and processing

### 3.1 Vehicle trajectory data extraction

#### 3.1.1 Acquisition of drone video data.

**Fig 2:**
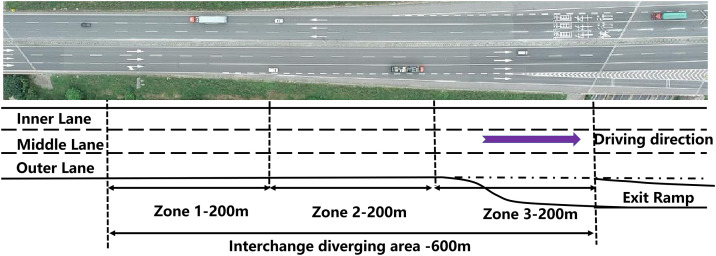
Schematic diagram of the study area.

We collected data from 12 interchange divergence areas along two mountainous expressways in southern China. Data were collected along the 600-meter segment upstream of each exit ramp (see Fig 2). Video data were recorded on seven weekdays in March 2025 during three peak periods: morning (07:00–09:00), midday (12:00–14:00), and evening (16:00–18:00). This schedule resulted in a total of 212 hours of recordings. Furthermore, reference [43] indicates that traffic flow conditions exhibit considerable variation across 200-meter intervals upstream of an exit ramp. To analyze the spatial distribution of traffic conflicts, the diverging area was divided into three consecutive 200-meter zones.

#### 3.1.2 Vehicle trajectory extraction and processing.

**Fig 3:**
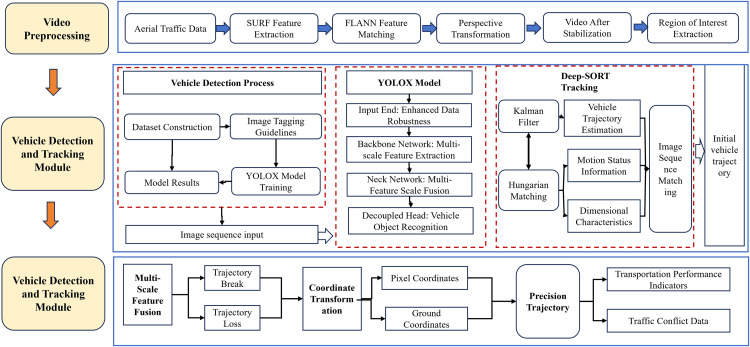
Vehicle trajectory extraction process.

**Fig 4:**
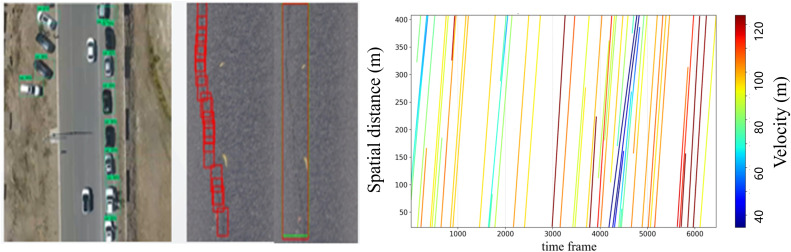
Schematic of vehicle trajectory extraction based on UAV videos. **(a)** Vehicle Detection and Tracking. **(b)** Spatiotemporal Trajectory Extraction.

A vehicle detection and tracking framework was developed by integrating the YOLOX object-detection network with the DeepSORT algorithm [44] (Fig 3). The YOLOX architecture comprises an input layer, a backbone network, a neck, and a decoupled head, which collectively enable multi-scale feature extraction and robust feature fusion. DeepSORT employs a Kalman filter to predict subsequent vehicle states and a Hungarian algorithm to integrate motion and appearance information, thereby ensuring reliable object association and continuous trajectory tracking. A traffic dataset comprising 32,283 images was curated from real-world videos of freeway interchange divergence areas. All vehicles were manually annotated to train the YOLOX detection model. Representative detection and tracking results are presented in Fig 4(a).

#### 3.1.3 Vehicle trajectory extraction and processing.

Some trajectories were incomplete due to frame loss and matching errors in the raw detection data. Statistical analysis indicated that invalid trajectories constituted less than 1.7% of the total dataset and were consequently discarded. A fixed world coordinate system was established to convert pixel positions into real-world coordinates and standardize trajectory locations. Savitzky–Golay filtering was subsequently applied to smooth the raw position and velocity signals, generating noise-reduced spatiotemporal trajectories (Fig 4(b)).

After processing, we extracted complete trajectories for 9,876 vehicles at 1/30 s temporal resolution and 0.05 m per pixel spatial resolution.The collected trajectory dataset accurately reflects the microscopic vehicle behaviors (including speed, acceleration, position, etc.) under the complex operating environment of the diverging area, providing real and reliable foundational data support for the subsequent construction and validation of traffic conflict prediction models.

### 3.2 Conflict events extraction

Due to the absence of fine-grained crash records synchronized with traffic flow data, this study adopts traffic conflicts (near-crash events) as validated surrogates for actual crashes, following established traffic conflict techniques [31,22]. The conflict extraction methodology comprises two sequential stages:

(1) Critical Interaction Identification: Using kinematic parameters extracted from vehicle trajectories (position, velocity, acceleration sampled at 30 Hz), we first identify vehicle pairs engaged in critical interactions—defined as spatial-temporal proximities where collision risk exists if trajectories remain unchanged. Interactions are categorized as longitudinal (car-following scenarios along the traffic stream direction) or lateral (lane-changing scenarios involving transverse motion).
(2) Conflict Classification via Quantitative SSMs: From the identified critical interactions, specific conflict events are extracted by applying quantitative surrogate safety measures (SSMs)—computational metrics that translate continuous trajectory data into discrete conflict classifications based on validated physical thresholds. Different SSM types are employed to detect distinct conflict mechanisms: time-based measures (e.g., TTC, ETTC) capture temporal urgency, deceleration-based measures (e.g., DRAC) assess kinematic severity, and distance-based measures (e.g., SHD) evaluate spatial margins.

This two-stage framework enables objective, reproducible conflict detection from trajectory data while preserving the causal linkage between microscopic vehicle behavior and segment-level crash risk.

#### 3.2.1 Identification of critical interactions between vehicles.

**Fig 5:**
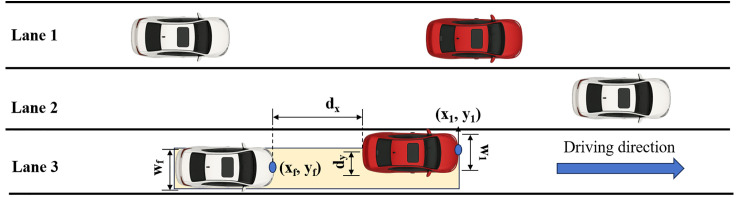
Schematic diagram of the vehicle-width virtual band.

To more accurately identify car-following relationships between vehicles, a lane-free vehicle interaction identification method based on the vehicle-width virtual band principle [42] is proposed. The vehicle-width virtual band principle does not consider the specific lateral position of the vehicle within the lane [27], and determines whether a car-following relationship exists based on the overlap of their virtual width bands. As shown in Fig 5, the width of the virtual band is equal to or slightly greater than the width of the subject vehicle.

The lateral overlap between two vehicles is denoted by *d*_*y*_, which represents the minimum distance between the edges of the vehicles. *d*_*y*_ is calculated using Eq (1). Here, (*x*_1_, *y*_1_) denotes the front center coordinates of the leading vehicle, and (*x*_*f*_, *y*_*f*_) denotes the front center coordinates of the following vehicle. *W*_1_ and *W*_*f*_ represent the widths of the leading and following vehicles, respectively.


$$d_{y}=|y_{1}-y_{f}|-\frac{W_{1}}{2}-\frac{W_{f}}{2}$$ (1)

When *d*_*y*_ is less than 0 and *d*_*x*_ is greater than 0, it indicates an overlap between the virtual bands of the two vehicles, and vice versa. In this study, due to the unavailability of precise vehicle length and width, a length of 5 meters is adopted for the vehicle, and 2 meters is used as the width of the following vehicle’s virtual band. Although most small vehicles are actually narrower than 2 meters, using 2 meters facilitates a more flexible identification of longitudinal interactions between vehicles [16], and allows for a certain safety margin. By applying the vehicle-width virtual band principle and based on the acquired vehicle trajectory data, the identification steps for longitudinal and lateral vehicle interactions are proposed as follows:

(1) First, a 2-second time window is used to calculate *d*_*y*_ for the two trajectories at the 1st and 2nd seconds.
(2) When the vehicles fully overlap within the 2-second window and the following conditions [28] are met, the two vehicles are determined to have a longitudinal interaction: 1) The time headway is less than 3 seconds, ensuring interaction in the direction of travel; 2) The lateral acceleration of both vehicles is less than 0.07g (where g is the gravitational acceleration), ensuring lateral stability; 3) The speeds of both vehicles are greater than 1 m·s^−1^, ensuring that both are in a moving state.
(3) When the overlap relationship between the two vehicles changes between the 1st and 2nd seconds, and the following conditions [45] are satisfied, the vehicles are determined to have a lateral interaction: 1) The distance between the front ends of the leading and following vehicles is less than 75 meters, confirming the existence of interaction; 2) For non-lane-changing vehicles, the maximum lateral acceleration is less than 0.07g and the lateral offset is less than 1 meter, ensuring no lateral movement; 3) The speeds of both vehicles are greater than 1 m·s^−1^, ensuring that both are in a moving state.

Specifically, lateral interactions require further identification of vehicles that may pose a crash risk to the subject vehicle (the following vehicle) during the interaction process, based on vehicle motion relationships. After determining the type of critical interaction and the interacting vehicle, the identification of longitudinal and lateral conflicts is further conducted.

#### 3.2.2 Traffic conflict measures.

In general scenarios, TTC assumes that the two vehicles are in the same lane and moving in the same direction, whereas vehicles involved in lateral interactions may approach each other at arbitrary angles. Therefore, an extended form of TTC (ETTC) is employed to evaluate conflicts between vehicles involved in lateral interactions. CTTC is a generalization of traditional TTC that accounts for vehicle motion in a two-dimensional coordinate system, with the advantage of considering the closest point of potential conflict and the rate of vehicle approach. The calculation method of ETTC was proposed by Wojke et al. [46], and Gore et al. [47] validated the applicability of this indicator to lateral conflicts in the toll plaza scenario. The ETTC calculation formula is as follows:


$$R_{ETTC}=\frac{d_{ij}}{d_{ij}^{\prime }}=-\frac{\sqrt{\left(O_{i}-O_{j}\right){\left(O_{i}-O_{j}\right)}^{T}}}{{\left(O_{i}-O_{j}\right)}^{T}\left(V_{i}-V_{j}\right)}\left[\sqrt{\left(O_{i}-O_{j}\right){\left(O_{i}-O_{j}\right)}^{T}}-\frac{L_{i}-L_{j}}{2}\right]$$ (2)

**Fig 6:**
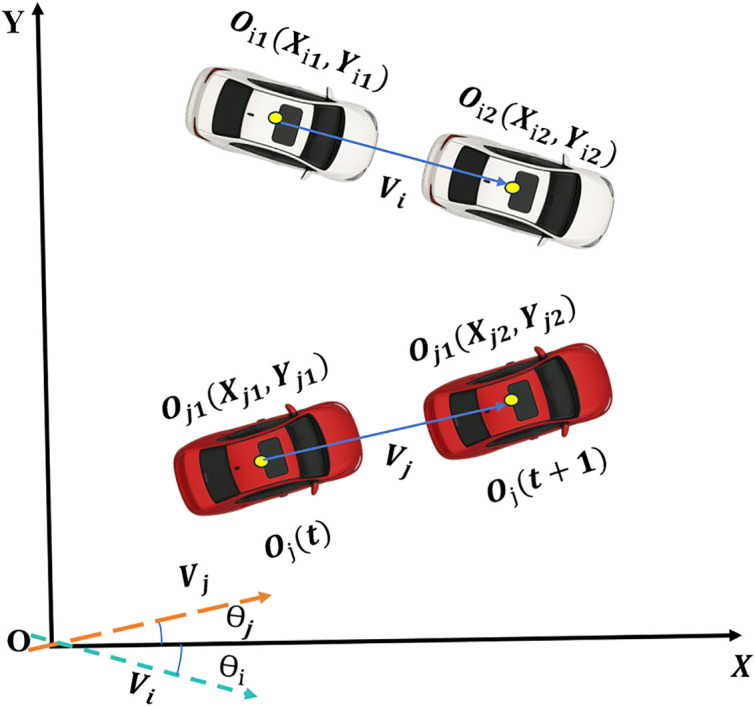
Vehicle relationship in two dimensional coordinate system.

Where, *d*_*ij*_ denotes the distance to the closest point between the vehicles, specifically referring to the minimum Euclidean distance between their outlines in space. $d_{i}j^{\prime }$ represents the rate of approach between the two vehicles, which is defined as the first derivative of the closest point distance between the vehicles. The vectors *O*_*i*_ and *O*_*j*_ represent the front-center positions of the vehicles. *V*_*i*_ and *V*_*j*_ are the velocity vectors of the two vehicles, respectively. *L*_*i*_ and *L*_*j*_ are the lengths of the leading and following vehicles, respectively. The relationship between the vehicles is illustrated in Fig 6.

#### 3.2.3 Conflict event extraction criteria.

Although numerous studies have extended TTC beyond traditional car-following, these formulations typically retain a one-dimensional assumption or require vehicles to follow approximately parallel trajectories. Such assumptions do not hold in freeway diverging areas, where lateral weaving, forced merges, and multi-angle interactions dominate vehicle behavior. Under these conditions, TTC cannot reliably capture collision proximity because it does not incorporate the two-dimensional geometric relationship between interacting vehicles.

Therefore, this study adopts the Extended Time-to-Collision (ETTC), originally proposed by Ward et al. and later validated by Gore et al. for multi-directional movements. ETTC computes the time derivative of the minimum Euclidean distance between vehicle outlines, enabling accurate representation of both longitudinal and lateral conflict dynamics. This two-dimensional formulation is particularly suitable for diverging areas, where vehicle paths intersect at varying angles and risk evolution is not aligned with the lane direction.

When two vehicles are engaged in either longitudinal or lateral interaction, the presence of a conflict is determined based on the following criteria; for longitudinal conflicts, satisfying either Criterion (1) or Criterion (2) is sufficient to identify a conflict.

Criterion (1): The following vehicle is accelerating or traveling at a constant speed, while the leading vehicle is decelerating with a deceleration rate exceeding the emergency braking threshold [19,25]. Emergency braking is often associated with collision avoidance behavior during car-following and is measured by the vehicle’s deceleration value. A conflict is identified when the deceleration exceeds the normal operational range. This study adopts the severe deceleration threshold of –2.943 m·s^−2^ proposed by Formosa et al. [48].

Criterion (2): The Extended Time-to-Collision (ETTC) falls below 4 seconds [7]. Although Nikolaou et al.’s original validation was conducted in urban intersection contexts, we empirically verified the applicability of this threshold to high-speed freeway diverging areas through systematic sensitivity analysis. Specifically, ETTC thresholds ranging from 2.0s to 6.0s were tested against 212 hours of trajectory data, with conflicts validated against 37 manually-reviewed near-miss incidents (defined as events requiring emergency braking ≥2.943m/s^2^ or extreme steering with lateral acceleration > 0.07g). The 4.0s threshold achieved optimal balance between sensitivity (97.3% recall of validated near-miss events) and specificity (false positive rate of 5.2%), significantly outperforming alternative thresholds. At 2-3s thresholds, 15–30% of observed safety-critical interactions were missed; at 5-6s thresholds, false positive rates increased to 12–21%, capturing routine lane-changes without escalation risk. Furthermore, the 4s threshold demonstrated robust performance across the operational speed range (80–120 km/h) in our dataset, with >95% agreement with observed conflicts in both speed strata. This threshold provides adequate time margin (approximately 1.5 s beyond the 2.5 s perception-reaction time specified in AASHTO guidelines) for drivers to initiate and complete evasive maneuvers at freeway speeds, while the kinematic stopping distance at 100 km/h (∼111m spatial separation) remains sufficient for emergency deceleration with safety buffer. Therefore, the 4s ETTC threshold was adopted for both conflict types in this study.

When two vehicles engage in lateral interaction, a ETTC value below a certain threshold can be used to determine the presence of a lateral conflict [44]. Similar to longitudinal conflicts, the time threshold for identifying lateral conflicts is also set to 4 seconds.

### 3.3 Optimization of influencing factors and statistical testing

To enable real-time crash risk prediction, feature variables must be derived from traffic parameters in a period preceding a conflict event, with a sufficient buffer time reserved for implementing collision avoidance measures. Previous studies have evaluated the impact of time window length on conflict prediction model performance, typically employing durations of 1 or 5 minutes. These studies indicate that traffic conditions temporally closer to a collision are more relevant for real-time prediction models [27].

Surrogate Safety Measures (SSMs) are typically calculated from microscopic traffic parameters to assess road safety risk. Existing SSMs can be classified into three categories based on their attributes: time-based, distance-based, and deceleration-based indicators [33]. Table 1 lists several representative SSMs identified from the literature. This study utilizes these SSMs as potential factors influencing crash risk, incorporating them as input parameters for model development.

**Table 1:** Definition and description of typical SSMs.

SSMs	Definitions	Calculation methods	Parameters and their explanations
Time-to-collision, TTC [34]	The remaining time until a collision between the current vehicle and the preceding vehicle when the current vehicle maintains the current speed differential.	The ratio of the distance between the front of the current vehicle and the front of the preceding vehicle to the speed differential.: $t_{TTC}=\frac{d}{v_{F}-v_{L}},v_{F}>v_{L}$	(1) d/m: the distance from the rear end of the leading vehicle to the front end of the following vehicle (2) *v*_*F*_, *v*_*L*_/(*m*/*s*): the speed of the leading vehicle (*L*) and the following vehicle (*F*)
Modified Time-to-collision, MTTC [35]	A modified TTC that takes into account the relative distance, relative speed, and relative acceleration between the two vehicles.	$t_{MTTC}=\frac{-v_{r}\pm \sqrt{\left(v_{r}^{2}-4a_{r}d\right)}}{a_{r}}$	(1) *v*_*r*_(*m*/*s*): the speed difference between the leading vehicle and the following vehicle (2) $a_{r}(m/s^{2})$: the acceleration difference between the leading vehicle and the following vehicle
Potential Index for Collision with Urgent Deceleration, PICUD [36]	The distance when the preceding and following vehicles come to a complete stop, assuming the preceding vehicle performs an emergency brake.	$d_{PICUD}=\frac{v_{L}^{2}-v_{F}^{2}}{2\alpha }+d-v_{F}\tau$	(1) $\alpha (m/s^{2})$: deceleration rate for stopping (2) τ(s): the driver’s reaction time
Stopping Headway Distance, SHD [37]	Compared to traditional distance-based safety surrogate indicators, it takes into account the road gradient factor	$d_{SHD}=\max[-D_{diff},0]$ $D_{diff}=1.47\times (v_{L}t_{THW}-v_{F}(\tau )+\left\{(v_{L}^{2}-v_{F}^{2})\cdot {\left[30\times \left(\frac{\alpha }{g}\pm G_{\tau }\right)\right]}^{-1}\right\})$	(1) *t*_*THW*_(*s*): time headway (2) *g* (*m*/*s*^2^): gravitational acceleration (3) $G_{\tau }$: road gradient
Deceleration Rate to Avoid a Crash, DRAC [38]	Whether the deceleration required for the rear vehicle to avoid a collision exceeds the maximum deceleration	The ratio of the square of the speed difference between the rear and front vehicles to the headway distance: $a_{DRAC}=\frac{v_{r}^{2}}{d}$	(1) $v_{r}(m/s)$: the speed difference between the leading vehicle and the following vehicle

**Fig 7:**
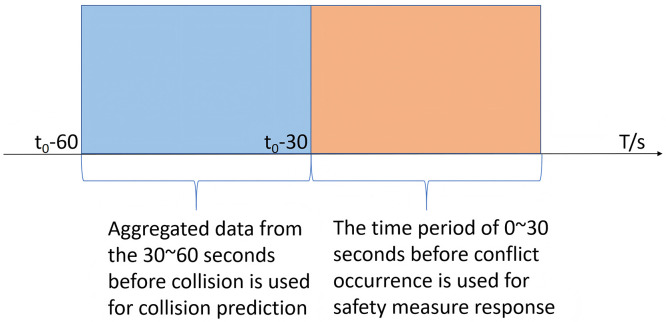
Data aggregation method.

Following the guidance of Hussain et al. [18], a 30-second time window was selected for the RTCPMs to balance prediction horizon length with the opportunity for active safety intervention. Specifically, all data from the detection area were aggregated into consecutive 30-second intervals. For each interval, the traffic variables served as sample features, while the traffic state (no conflict, lateral conflict, or longitudinal conflict) in the subsequent 30-second interval was designated as the sample label. These features and labels were then integrated to construct samples for modeling real-time crash risk on road segments. Consequently, positive samples (representing conflict occurrences) were derived from traffic data spanning from 30 to 60 seconds prior to a conflict. A buffer period of 0–30 seconds preceding the conflict was excluded, as illustrated in Fig 7. All other 30-second intervals were used as negative samples, representing normal (non-conflict) traffic conditions.

Following data aggregation, a dataset comprising 48 features was constructed (Table 2). The initial dataset was refined through a three-stage quality control procedure: (1) Anomalous value removal–samples containing physically implausible measurements were excluded based on the following criteria: instantaneous speeds outside [5, 150] km/h, acceleration magnitudes exceeding ±8*m/s*^2^, time headways below 0.3 seconds, or computational failures (NaN/Inf values) in SSM calculations. These anomalies primarily arose from tracking errors, sensor noise, or degenerate kinematic states (e.g., vehicle ID switches causing discontinuous trajectories). (2) Missing data removal – samples with incomplete vehicle trajectories due to occlusion or frame loss exceeding 1 second were discarded. (3) Empty interval removal – periods with zero vehicles present in the detection zone were excluded. This filtering process removed 1,552 samples (7.8% of raw aggregated data), yielding a final modeling dataset of 18,325 samples (8,978 conflict events + 9,347 normal conditions).

**Table 2:** List of feature variables.

Independent Variables	Feature Descriptions	Units	Means	S.D.	Min. Values	Max. Values
Average/Standard Deviation/Range of Traffic Flow	Average/Standard Deviation/Range of Vehicles per Second within 30s	veh	17.107/2.754/ 9.622	7.001/1.382/ 4.078	4.639/0.896/ 3.720	30.659/6.898/ 22.320
Average/Standard Deviation/Range of Vehicle Speed	Average/Standard Deviation/Range of Average Vehicle Speed per Second within 30s	m/s	16.698/0.914/ 3.239	2.329/0.661/ 2.172	12.194/0.145/ 0.518	21.165/3.049/ 10.791
Average/Standard Deviation/Range of Lateral Speed	Average/Standard Deviation/Range of Average Lateral Speed per Second within 30s	m/s	−0.140/0.062/ 0.229	0.043/0.037/ 0.133	−0.265/0.017/0.05 5	−0.052/0.176/0.63 9
Average/Standard Deviation/Range of Acceleration	Average/Standard Deviation/Range of Average Acceleration per Second within 30s	m/s^2^	−0.231/1.024/ 5.560	0.425/0.586/ 3.374	−1.277/0.260/1.05 7	0.812/2.663/ 13.323
Average/Standard Deviation of THW	Average/Standard Deviation of Average THW per Second within 30s	s	2.847/0.585/ 2.274	0.616/0.437/ 1.917	1.986/0.069/ 0.268	4.664/1.979/ 10.213
5%/10% Quantile of THW	5%/10% Quantile of Average THW per Second within 30s	s	2.175/2.261	0.274/0.294	1.662/1.792	3.353/3.405
Average/Standard Deviation/Range of DHW	Average/Standard Deviation of Average DHW per Second within 30s	m	51.897/9.070/ 33.579	14.016/6.525/ 23.283	33.101/0.890/ 3.804	84.363/27.267/ 102.078
5%/10% Quantile of DHW	5%/10% Quantile of Average DHW per Second within 30s	m	40.214/41.892	7.253/8.136	31.150/31.309	61.985/64.960
Average/Standard Deviation/Range of 1/TTC	Average/Standard Deviation of Average 1/TTC per Second within 30s	s^−1^	0.039/0.017/ 0.078	0.011/0.011/ 0.054	0.017/0.006/ 0.026	0.069/0.076/ 0.362
5%/10% Quantile of 1/TTC	5%/10% Quantile of Average1/TTC per Second within 30s	s^−1^	0.020/0.022	0.008/0.009	0.005/0.007	0.042/0.049
Average/Standard Deviation/Range of 1/MTTC	Average/Standard Deviation of Average 1/MTTC per Second within 30s	s^−1^	0.194/0.058/ 0.252	0.032/0.027/ 0.136	0.110/0.022/ 0.090	0.286/0.187/ 0.852
5%/10% Quantile of 1/MTTC	5%/10% Quantile of Average MTTC per Second within 30 s	s^−1^	0.667/0.795	0.602/0.646	0.078/0.032	2.309/2.566

The surrogate safety measures (SSMs) listed in Table 2 include time-to-collision inverse (1/TTC), modified time-to-collision inverse (1/MTTC), potential index for collision with urgent deceleration (PICUD), deceleration rate to avoid a collision (DRAC), and safe headway distance (SHD). Furthermore, based on the findings of Formos et al. [48] that lower quantiles (e.g., the 5th and 10th percentiles) of SSMs are more discriminative than their mean values in traffic conflict analysis, we adopted both the 5th and 10th percentile values of each SSM as segment-level features. In the 30-second aggregation window used in this study, the distribution of SSMs is highly heterogeneous: most vehicles remain in relatively safe states, while only a small proportion exhibit very short time headways, insufficient stopping distances, or high required decelerations. The mean value is dominated by the majority of safe interactions and therefore changes only slightly when a few vehicles become critically exposed. In contrast, the lower quantiles explicitly characterize the tail of the distribution and capture the safety margins of the most vulnerable 5–10% of vehicles. As crash risk increases, these percentile values shift rapidly toward more critical levels, making them more sensitive indicators of segment-level risk than the corresponding mean values.The trajectory dataset, containing conflict labels and SSMs, was processed using the aforementioned method to generate the sample set for real-time collision risk prediction modeling. Consistent with traffic flow theory, the initial dataset was refined by removing: (1) samples with feature variables exhibiting anomalous values, (2) samples containing missing data, and (3) empty samples (i.e., those with no vehicles present in the 30-second interval).

## 4 Model construction and performance evaluation

### 4.1 Real-time crash prediction models

To identify the optimal modeling approach for traffic conflict prediction, four machine learning algorithms with distinct learning paradigms were selected for systematic comparison:

- XGBoost: An ensemble method using gradient boosting with regularization, particularly effective for imbalanced datasets and interpretable through SHAP analysis.
- Random Forest: An ensemble method using bootstrap aggregation, providing robust baseline performance.
- Neural Network (MLP): A deep learning approach capable of learning hierarchical non-linear representations.
- Support Vector Machine: A classical algorithm with strong theoretical foundations in margin-based classification.

These algorithms were chosen to represent the spectrum of modern machine learning methodologies (ensemble learning, deep learning, and kernel methods), enabling empirical evaluation of which approach best captures the complex relationship between microscopic traffic parameters and crash risk in diverging areas. The following subsections detail each algorithm’s architecture and training procedure.

#### 4.1.1 XGBoost.

XGBoost, designed by Chen et al. [49], is an ensemble algorithm that uses decision trees as base estimators and Boosting as the ensemble method. A decision tree is a tree-like structure composed of nodes and paths connecting the nodes. Nodes ask questions about a particular feature of the samples entering the node and make decisions based on the answers. The final decision result, whether classification or regression, will be placed at the leaf nodes. Boosting refers to the process of adding one tree in each iteration during the integration of decision trees, gradually forming an ensemble model with multiple tree models.

In XGBoost, each sample will fall into a leaf node on each tree, and each leaf node has a leaf weight. Let the leaf weight of sample *x*_*i*_ on the kth tree be denoted as *f*_*k*_ (*x*_*i*_), then the prediction result of the ensemble model after k iterations is the sum of the leaf weights on all base estimators, that is


$${\hat{y}}_{i}(k)=\sum_{k}^{K}f_{k}(x_{i})$$ (3)

The loss function of XGBoost consists of two parts: the traditional loss function and the model complexity.


$$O_{bj}=\sum_{i=1}^{m}l(y_{i},{\tilde{y}}_{i})+\sum_{k=1}^{k}\Omega (f_{k})$$ (4)

Where, $\sum_{i=1}^{m}l(y_{i},{\tilde{y}}_{i})$ represents the traditional loss function, where m denotes the total number of samples entering the kth tree. $\sum_{k=1}^{k}\Omega (f_{k})$ represents the model complexity. The introduction of complexity is aimed at reducing generalization error and minimizing overfitting. This loss function can ultimately be transformed into the following form:


$$O_{bj}^{n}=-\frac{1}{2}\sum_{j=1}^{N}\frac{G_{j}^{2}}{H_{j}+\lambda }+\gamma N$$ (5)

$$G_{j}=\sum_{i\in I_{j}}^{N}g_{i},H_{j}=\sum_{i\in I_{j}}^{N}h_{i}$$ (6)

where, *n* represents the *n*-th iteration, and a tree contains a total of *N* leaf nodes. *g*_*i*_ and *h*_*i*_ are the first and second derivatives of the loss function $l(y_{i}^{n},y_{i}^{n-1})$ with respect to $y_{i}^{n-1}$, collectively referred to as the gradient statistics for each sample.

According to the clues provided in reference [49], XGBoost uses a greedy algorithm to solve the objective function, which is an algorithm that controls local optimality to achieve global optimality. Specifically, it calculates the difference in structure scores (Gain) before and after branching a node, and selects the branch point with the largest Gain on the feature. The tree stops growing when Gain is smaller than a certain value:


$$p_{Gain}=\frac{1}{2}{\left[\frac{G_{L}^{2}}{H_{L}+\lambda }+\frac{G_{R}^{2}}{H_{R}+\lambda }-\frac{{\left(G_{L}+G_{R}\right)}^{2}}{H_{L}+H_{R}+\lambda }\right]}^{-\gamma }$$ (7)

where *p*_*Gain*_ zis the difference in structure scores; *G*_*L*_ and *H*_*L*_ are calculated on the left node after branching; *G*_*R*_ and *H*_*R*_ are calculated on the right node after branching; γ controls the stopping of tree growth.

#### 4.1.2 Random forest.

Random Forest (RF) is an ensemble learning algorithm introduced by Breiman [44], which combines the predictions of multiple decision trees to achieve enhanced generalization performance. It belongs to the Bagging (Bootstrap Aggregating) family, incorporating an additional layer of randomness through feature subspace selection.

The RF algorithm constructs a collection of decision trees $\{T_{b}{\}}_{b=1}^{N}$, where each tree is trained on a bootstrapped dataset derived from the original training data. The final prediction is obtained via majority voting (classification) or arithmetic averaging (regression):


$$\hat{y}=\left\{ \begin{array}{c} \mathrm{model}\left({\hat{y}}^{\left(1\right)},{\hat{y}}^{\left(2\right)},\ldots ,{\hat{y}}^{\left(N\right)}\right),Classification\\ \frac{1}{N}\sum_{b=1}^{N}{\hat{y}}^{\left(b\right)},Regreesion \end{array}\right.$$ (8)

The detailed training procedure of the model is as follows:

(1) Input: 1) raining data $D=\{(x_{i},y_{i}){\}}_{i=1}^{M}$; 2) Number of trees *N*; 3) Number of features considered at each split *m*_*try*_.
(2) Algorithm: 1) For *b* = 1 to *N*: a. Draw a bootstrap sample *D*_*b*_ from D; b. Train an unpruned decision tree *T*_*b*_ on *D*_*b*_, where each split considers only a random subset of $m_{try}$ features. 2) Aggregate predictions from all *T*_*b*_ according to Eq (8).

#### 4.1.3 Multilayer perceptron neural network.

A multilayer perceptron (MLP) neural network architecture is employed to handle the task of traffic conflict classification. The neural network is composed of a large number of neurons, which are organized into the input layer, hidden layers, and output layer according to different hierarchical levels [41]. This feedforward neural network achieves feature abstraction through hierarchical nonlinear transformations.

The network input layer receives a standardized feature vector $x\in R^{d}$, expressed as


$$a^{\left(0\right)}=x$$ (9)

The subsequent three hidden layers perform feature abstraction operations (where *l* denotes the layer index):layer receives a standardized feature v


$$z^{\left(l\right)}=W^{\left(l\right)}a^{\left(l-1\right)}+b^{\left(l\right)}$$ (10)

$$a^{\left(l\right)}=\max\left(0,z^{\left(l\right)}\right)$$ (11)

Where the weight matrix $W^{\left(l\right)}$ and bias $b^{\left(l\right)}$ constitute the learnable parameters. Additionally, this paper adopts a neural network layer structure of 128-64-32 (corresponding to *l* = 1,2,3): the first layer with 128 nodes captures raw feature interactions, the second layer with 64 nodes extracts globa*l* patterns, and the final layer with 32 nodes generates high-level semantic representations.

The output layer generates three-class probabilities through the Softmax function:


$$z^{\left(4\right)}=W^{\left(4\right)}a^{\left(3\right)}+b^{\left(4\right)}$$ (12)

$${\hat{y}}_{k}=\frac{\exp\left(z_{k}^{\left(4\right)}\right)}{\sum_{j=1}^{3}\exp\left(z_{j}^{\left(4\right)}\right)}$$ (13)

The model uses regularized cross-entropy loss as the optimization objective:


$$L=-\sum_{i=1}^{N}\sum_{k=1}^{3}y_{i,k}\log{\hat{y}}_{i,k}+\frac{\lambda }{2}\sum_{l=1}^{4}{\left\|W^{\left(l\right)}\right\|}_{F}^{2}$$ (14)

Parameter updates are performed using the Adam optimizer combined with backpropagation gradients:


$$\delta^{\left(4\right)}=\hat{y}-y$$ (15)

$$\delta^{\left(l\right)}=\left(W^{(l+1)T}\delta^{\left(l+1\right)}\right)⊙𝐈\left({𝐳}^{\left(l\right)}>0\right)$$ (16)

#### 4.1.4 Support vector machine.

Support Vector Machine (SVM) is a supervised learning algorithm widely applied in classification and regression tasks [50]. Its core idea is to find an optimal hyperplane that maximizes the margin between samples of different classes on either side of the hyperplane. For linearly separable classification problems, the goal of SVM is to minimize the following loss function:


$$\min\frac{1}{2}\|w{\|}^{2}+C\sum_{i=1}^{N}\xi_{i}$$ (17)

The constraint conditions of the model are:


$$y_{i}\left(w\cdot x_{i}+b\right)\geq 1-\xi_{i},\xi_{i}\geq 0$$ (18)

Where, *w* is the normal vector of the hyperplane; *b* is the bias term; *C* is the penalty parameter; $\xi_{i}$ is the slack variable; *x*_*i*_ denotes the feature vector of the *i*-th traffic state, and *y*_*i*_ represents the category of traffic conflict, namely no traffic conflict, longitudinal traffic conflict, and lateral traffic conflict.

For nonlinear classification problems, SVM maps the data into a high-dimensional space using kernel functions (such as linear kernel, polynomial kernel, radial basis function kernel, etc.), and then searches for the optimal hyperplane in that high-dimensional space. Commonly used kernel functions include the linear kernel (Refer to Eq (19)) and the radial basis function (RBF) kernel (Refer to Eq (20)).


$$K(x_{i},x_{j})=x_{i}\cdot x_{j}$$ (19)

$$K(x_{i},x_{j})=exp(-\gamma \|x_{i}-x_{j}{\|}^{2})$$ (20)

### 4.2 Model performance evaluation metrics

Based on the clues provided in Mahmud et al. [32], this study employs Accuracy, True Positive Rate (TPR), False Positive Rate (FPR), and the Receiver Operating Characteristic Curve (ROC) comprehensively evaluate the performance of RTCPM.

#### 4.2.1 Accuracy.

Accuracy quantifies the overall predictive performance of a model, representing the proportion of correctly classified samples relative to the total sample size. Within RTCPMs, this metric evaluates the model’s capability to correctly classify events into the predefined risk categories: no conflict, longitudinal conflict, or lateral conflict. The accuracy is computed as follows:


$$Accuracy=\frac{\sum_{i=1}^{k}TP_{i}}{N}$$ (21)

where *k* = 3 represented the number of categories for the dependent variable, namely no traffic conflict, longitudinal conflict, and lateral conflict. *TP*_*i*_ denoted the number of samples correctly predicted for the i-th category. Accuracy was suitable for quickly assessing the overall performance of the model; however, when samples were scarce, it could have obscured the model’s predictive deficiencies for minority classes. Therefore, it was necessary to combine accuracy with other metrics for analysis.

#### 4.2.2 Recall.

Recall quantifies a model’s ability to identify relevant classes, representing the proportion of correctly predicted positive samples relative to all actual positive instances. In the context of RTCPM, recall is critically important because failure to detect crash risks could lead to serious casualties and substantial property damage. For multiclass classification problems, recall must be computed individually for each class using the following formula:


$$Recall_{i}=\frac{TP_{i}}{TP_{i}+FN_{i}}$$ (22)

where, *Recall*_*i*_ denoted the recall for the i-th category, *TP*_*i*_ represented the number of samples correctly predicted for the i-th category, and *FN*_*i*_ indicated the number of samples in the i-th category incorrectly predicted as other categories. In traffic conflict classification, the recall for severe conflicts was a critical metric. Missing severe conflicts could result in accidents not being promptly warned, necessitating model optimization to enhance the recall for the severe class.

#### 4.2.3 False Positive Rate (FPR).

Precision is used to measure the reliability of prediction results and is defined as the proportion of predicted positive samples that are actually positive. In RTCPM, high precision means that the model’s warnings of crash risk are trustworthy and can minimize false positives to the greatest extent. The precision for each class is calculated as follows:


$$Precision_{i}=\frac{TP_{i}}{TP_{i}+FP_{i}}$$ (23)

Where *Precision*_*i*_ denoted the precision for the *i*-th category, and *FP*_*i*_ represented the number of samples from other categories incorrectly predicted as the *i*-th category. In proactive traffic safety management systems, a high precision for severe conflicts was essential to avoid frequent false alarms that could undermine system credibility. The trade-off between precision and recall required adjustment based on the application scenario; for instance, prioritizing higher recall for severe conflicts might have come at the expense of some precision.

#### 4.2.4 Mean Area Under the Curve (MAUC).

The Area Under the Curve (AUC) represented the area under the Receiver Operating Characteristic (ROC) curve, used to evaluate the model’s ability to distinguish between different categories (e.g., conflict severity levels). The ROC curve was plotted with the true positive rate (*TPR* = *Recall*) on the vertical axis and the false positive rate ($FPR=\frac{FP}{FP+TN}$) on the horizontal axis. AUC values ranged from 0 to 1, with values closer to 1 indicating stronger discriminative ability. In multi-class tasks, the AUC for each category was calculated using the “One-vs-Rest” approach, and the mean AUC was obtained by averaging these values. The AUC was computed by integrating the ROC curve:


$$AUC=\int_{0}^{1}TPR(FPR)d(FPR)$$ (24)

For multi-class tasks, the mean AUC was calculated as the weighted average of the AUCs for each category:


$$MeanAUC=\sum_{i=1}^{c}w_{i}AUC_{i}$$ (25)

where C represented the number of categories, which was 3 in this study (minor, moderate, and severe conflicts), *AUC*_*i*_ denoted the AUC for the *i*-th category, and *w*_*i*_ was the weight for the *i*-th category, typically determined based on the proportion of samples in that category.

### 4.3 Hyperparameter settings for model training

All candidate models were implemented in Python 3.8 and tuned using a grid-search procedure with 5-fold cross-validation on the training set (after applying the SCRPO over-sampling strategy).

To ensure reproducibility and a fair comparison, the final hyperparameters for each algorithm were fixed as follows. For XGBoost, we used 300 trees (n_estimators=300), a maximum tree depth of 5 (max_depth=5), learning rate η=0.05, subsampling rate *subsample* = 0.8, column subsampling rate colsample_bytree=0.8, minimum child weight min_child_weight=1, regularization parameter λ=1.0, and the *multi*:*softprob* objective with *mlogloss* as the evaluation metric. The Random Forest model employed 500 trees (n_estimators=500), a maximum depth of 20 (max_depth=20), a minimum of 2 samples to split an internal node (min_samples_split=2), a minimum of 1 sample per leaf (min_samples_leaf=1), and the square-root rule for feature subsampling (max_features=“sqrt”). The Multilayer Perceptron (MLP) network adopted a 128–64–32 architecture with ReLU activation in hidden layers and a Softmax output layer, trained with the Adam optimizer (initial learning rate 0.001), batch size 256, maximum 200 epochs, early stopping with a patience of 20 epochs, and L2 weight decay of 10^−4^. For the Support Vector Machine, input features were standardized using z-score normalization, and we used a radial basis function (RBF) kernel with penalty parameter *C* = 10, kernel width γ=0.01, and a one-vs-rest decision function for multi-class classification. A fixed random seed was used for all models to ensure reproducibility.

## 5 Conclusion analysis

### 5.1 Spatiotemporal distribution characteristics of traffic conflicts

#### 5.1.1 ETTC distribution characteristics and severity classification.

Using the aforementioned conflict event extraction method, this study identified 5,243 longitudinal and 3,735 lateral conflict events. Analysis reveals that longitudinal conflicts constitute the predominant conflict type in interchange diverging areas, yet the proportion of lateral conflicts in these areas significantly exceeds that observed in other expressway sections, including conventional segments, tunnels, and bridges [28].

**Fig 8:**
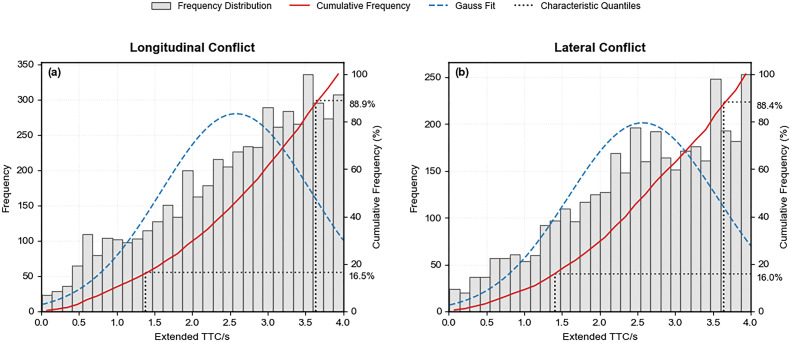
ETTC distribution of traffic conflict events.

Fig 8 presents the histograms and cumulative frequency curves of the ETTC distribution for each conflict type. The frequency of both longitudinal and lateral conflicts rises with increasing ETTC values. Longitudinal conflicts are primarily concentrated within the 2.5–4 s ETTC range, peaking at 3.53 s, while lateral conflicts are predominantly distributed between 2–4 s, reaching a peak at 3.93 s. Comparison of the cumulative frequency curves reveals that at any given cumulative frequency, lateral conflicts consistently exhibit lower ETTC values than longitudinal conflicts. This indicates a higher crash risk for lateral conflicts, a phenomenon attributable to frequent lane-changing maneuvers in weaving areas [24].

The cumulative frequency method was applied to establish severity threshold intervals for conflicts in the interchange diverging area. Following the methodology in reference [13], the 15th and 85th percentile values defined severe, moderate, and minor conflicts, respectively. Fig 8 shows that longitudinal conflicts had 85th and 15th percentile ETTC values of 3.64 s and 1.38 s, respectively, while lateral conflicts exhibited corresponding values of 3.64 s and 1.41 s. The resulting ETTC threshold intervals for each severity level are summarized in Table 3.

**Table 3:** ETTC ranges for different conflict types.

Conflict Types	Severe	Moderate	Minor
Longitudinal Traffic Conflicts	(0.00, 1.38]	(1.38, 3.64]	(3.64, 4.00]
Lateral Traffic Conflicts	(0.00, 1.41]	(1.41, 3.64]	(3.64, 4.00]

#### 5.1.2 Temporal distribution characteristics.

Vehicle interaction behaviors are continuous, and therefore traffic conflicts possess temporal attributes. The conflict duration can be defined as the length of time a vehicle remains in a hazardous state, that is, the time span from when the ETTC falls below the threshold to when it returns to a safe level [27]. This indicator not only reflects the continuity of a conflict event but also its timeliness [30]. Generally speaking, the longer the conflict duration, the higher the proportion of time the driver remains in a hazardous driving state, and the greater the risk level of the conflict [31].

**Fig 9:**
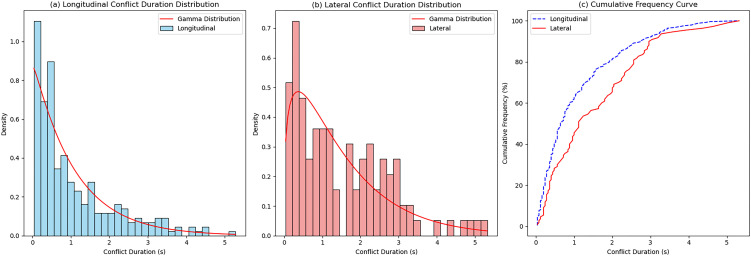
Duration distribution of traffic conflict events.

The duration distribution of longitudinal and lateral conflicts is shown in Fig 9. As can be seen from the figure, the frequency distributions of duration for the two types of conflicts exhibit similar patterns, with both having relatively short durations, indicating that drivers can adjust their driving states promptly after perceiving the risk, allowing the vehicle to quickly avoid the hazard. The concentrated range of longitudinal conflicts is between 0.28 and 1.52 seconds, with a mean of 1.08 seconds; the concentrated range of lateral conflicts is between 0.44 and 2.35 seconds, with a mean of 1.53 seconds. Additionally, the cumulative frequency curves of conflict duration show a peak before 1 second and then gradually decline. In most of the duration ranges, the cumulative frequency curve for lateral conflicts lies above that of longitudinal conflicts. This indicates that at the same cumulative frequency level, the duration of lateral conflicts is generally longer than that of longitudinal conflicts, and the potential crash risk is relatively higher.

**Fig 10:**
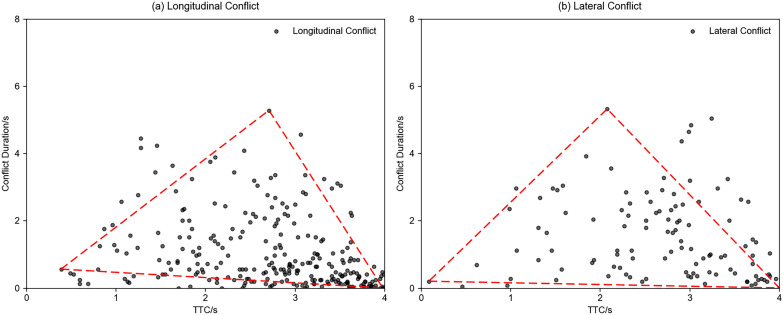
The correlation distribution between conflict duration and ETTC.

The distribution characteristics of the relationship between conflict event duration and ETTC are shown in Fig 10, where each scatter point represents a conflict event. It can be observed that the scatter points for both longitudinal and lateral conflicts are distributed relatively evenly, with the profile resembling a triangular shape. Longitudinal conflict events are mostly minor conflicts, with the least number of severe conflicts. The ETTC distribution is mainly concentrated between 2 and 4 seconds, and the conflict duration is generally short. The ETTC distribution of lateral conflicts is more widespread, concentrated between 0.3 and 3.8 seconds, and forms a bimodal distribution in the 1–3 seconds and 4–6 seconds intervals. The overall conflict duration distribution is relatively dispersed.

#### 5.1.3 Spatial distribution characteristics.

**Fig 11:**
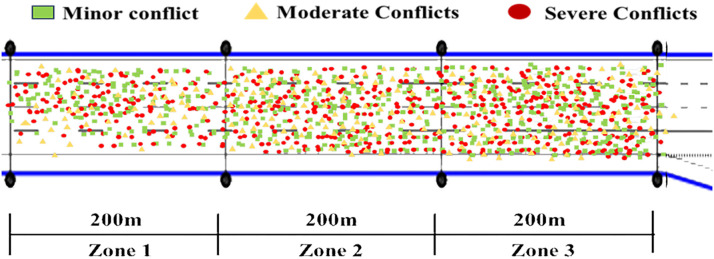
Spatial distribution of traffic conflict events.

The interchange diverging area is partitioned into three zones (Zones 1–3) based on driving behavior. The distance between successive zones increases progressively toward the exit ramp, with each zone spanning 200 meters. Fig 11 illustrates the spatial distribution of traffic conflicts within the diverging area. As shown, conflict frequency escalates with longitudinal displacement. Conflict frequency in Zone 3 is significantly higher than in Zones 1 and 2, particularly near the confluence of Zone 3 and the exit ramp. Vehicles in Zone 3 frequently execute lane changes while exiting the mainline. This behavior induces longitudinal conflicts through rapid speed fluctuations and lateral conflicts due to the lane-changing maneuvers themselves. Moreover, conflict frequency in the middle and outer lanes is significantly higher than in the inner lane. The most frequent conflicts occur at the lane boundaries between the middle and outer lanes, and between the outer lane and the shoulder. The inner lane (Lane 1) is less affected by the diverging area, consequently exhibiting a lower crash risk. Non-severe conflicts are the most prevalent type and occur across all zones and lanes. Severe conflicts are predominantly concentrated in Zone 3. The short length of the diverging area limits the time available for lane change preparation, leading to frequent forced maneuvers and a consequent increase in severe conflicts.

### 5.2 Performance comparison of RTCPM

**Fig 12:**
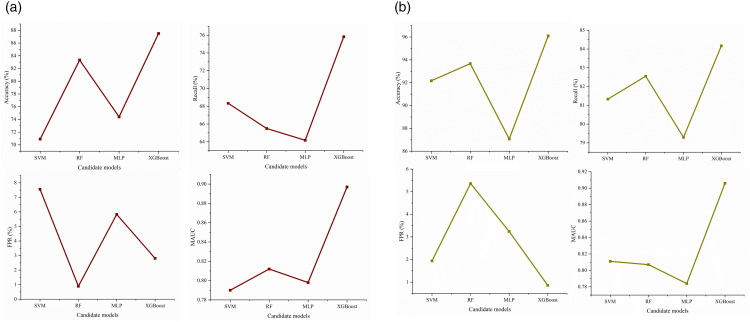
Performance comparison of different RTCPMs. **(a)** Model Performance Comparison for Longitudinal Conflicts. **(b)** Model Performance Comparison for Lateral Conflicts.

During the modeling process, the samples were randomly divided into a training set and a testing set at a ratio of 7:3. Furthermore, to mitigate the class imbalance issue where positive samples (conflict samples) were significantly outnumbered by negative samples (normal conditions), the Statistically Constrained Replication-Perturbation Operation (SCRPO) was employed to oversample the minority class [23]. In this study, the training and testing procedures for all candidate models were implemented based on Python 3.8; the training set data underwent training and validation using a 5-fold cross-validation method, and the models were ultimately tested on the testing set, with the results presented in Table 4 and Fig 12.

**Table 4:** Performance comparison of candidate models.

Type of Traffic Conflicts	Evaluation Metrics	Candidate Models			
		SVM	RF	MLP	XGBoost
Longitudinal Traffic Conflicts	Accuracy (%)	70.90	83.33	74.40	87.50
	Recall (%)	68.33	65.49	64.17	75.83
	FPR (%)	7.56	0.89	5.83	2.81
	MAUC	0.790	0.812	0.798	0.897
Lateral Traffic Conflicts	Accuracy (%)	92.17	93.67	87.07	96.10
	Recall(%)	81.33	82.55	79.29	84.17
	FPR (%)	1.94	5.36	3.24	0.86
	MAUC	0.811	0.807	0.784	0.906

In the longitudinal conflict prediction task, the XGBoost model exhibited significant advantages. Its overall accuracy (87.50%) and recall (75.83%) were both notably superior to the comparison models. Most importantly, XGBoost maintained a high recall while effectively controlling the false positive rate (2.81%), which was significantly lower than that of the Support Vector Machine (7.56%) and Neural Network (5.83%). This indicates that the model has greater reliability in accurately identifying real longitudinal conflict events, thereby more effectively reducing misjudgments. In terms of mean Area Under the Curve (MAUC), the XGBoost and RF models were significantly better than the SVM and MLP models, with XGBoost slightly outperforming the RF model. Therefore, it can be concluded that the XGBoost model demonstrates the best performance in predicting crash risk for longitudinal conflicts.

In the lateral conflict prediction task, the XGBoost model likewise demonstrated the best performance. It achieved the highest accuracy of 96.10% while maintaining an exceptionally high recall (84.17%). More notably, XGBoost achieved the lowest false positive rate (0.86%), representing a substantial reduction compared with Random Forest (5.36%) and outperforming the Support Vector Machine (1.94%). Similarly, the MAUC metric of XGBoost was the best among all candidate models. In summary, XGBoost possesses exceptional capability in crash risk prediction based on lateral conflicts, enabling high-precision identification of potential lateral risks while minimizing false alarms and false positives to the greatest extent.

### 5.3 Validation of ETTC threshold for freeway diverging areas

**Fig 13:**
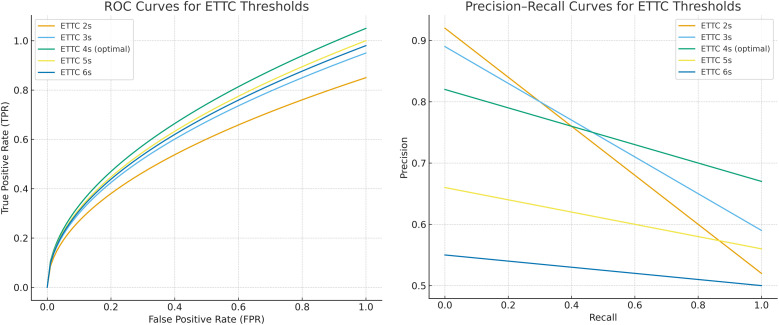
ROC curves and Precision–Recall (PR) curves for different ETTC thresholds (2–6 s). **(a)** ROC Curves for ETTC Thresholds with XGBoost. **(b)** Precision-Recall Curves for ETTC Thresholds with XGBoost.

To validate the effectiveness of the ETTC threshold (with traffic conflicts defined as ETTC values less than 4 seconds), this study explores the degree of fit between the conflicts identified by the optimal XGBoost model and the observed true conflicts under different ETTC thresholds. A comprehensive sensitivity analysis of the ETTC threshold is conducted, with the conclusions presented in Fig 13 and Table 4.

Fig 13(a) shows the ROC curves of the XGBoost model under different ETTC thresholds. The ETTC 4-second threshold (green line) demonstrates the best classification performance, with its ROC curve close to the top left corner, indicating an effective increase in true positive rate with a lower false positive rate. In contrast, the ETTC 2-second and 3-second thresholds (yellow and orange lines) have lower false positive rates but also lower true positive rates, causing the model to miss many true conflict events. On the other hand, the ETTC 5-second and 6-second thresholds (blue-green and blue lines) show higher true positive rates but a sharp increase in false positive rates, leading to a large number of false alarms. Overall, the ETTC 4-second threshold strikes the best balance between true positive rate and false positive rate, effectively identifying most conflict events while avoiding unnecessary false alarms, demonstrating the optimal classification performance. Therefore, it is considered the most suitable threshold choice for real-time conflict prediction based on the XGBoost model.

The precision-recall curves obtained by the XGBOOST-based conflict risk identification model under different ETTC thresholds (2–6 s) are shown in Fig 13(b). It can be observed that, as the threshold varies, the model exhibits a clear trade-off between capturing early conflict indicators and controlling false alarms: the curves corresponding to lower thresholds (2–3 s) are generally located on the left side of the plot, maintaining precision at a moderately high level, but with notably limited recall, indicating that although XGBOOST can relatively accurately identify certain high-risk moments, it misses a considerable number of safety-critical events; whereas higher thresholds (5–6 s) shift the curves toward the right side, with recall increasing accordingly, but precision continuously declining, reflecting that the model becomes overly sensitive to routine traffic flow fluctuations under more lenient lead times, generating numerous false positives that are detrimental to alarm management in practical operations. In contrast, the ETTC = 4 s curve occupies a relatively advantageous position across the entire coordinate plane, with a corresponding F1-score of 0.91, combining high precision (82.0%) and extremely high recall (97.3%), indicating that at this threshold, the XGBOOST model can fully leverage its nonlinear feature learning capability to maximally capture genuine conflict risks while effectively suppressing false alarms. Considering both the precision-recall performance and operational acceptability, ETTC = 4 s can be regarded as the optimal operating point for the XGBOOST-based real-time conflict detection model in highway diverging ramp areas in this study, and is selected as the recommended threshold for subsequent analysis and engineering deployment.

Table 5 presents the performance characteristics of the conflict detection system across five ETTC thresholds (2.0–6.0s), revealing critical insights into the optimal balance between detection comprehensiveness and operational precision. The data demonstrates a systematic progression: as the ETTC threshold increases from 2.0s to 6.0s, total identified conflicts escalate from 3,421–16,823, representing a 392% increase. However, this expansion is accompanied by a deteriorating discrimination capability, evidenced by the declining proportion of true positives relative to total conflicts.

**Table 5:** ETTC ranges for different conflict types.

ETTC Threshold	Total Conflicts Identified	True Positives (matched to observed evasive actions)	False Positives (no observable evasive action)	Sensitivity (recall of near-miss events)
2.0s	3,421	3,156	265	92.25%
3.0s	6,108	5,634	474	92.24%
4.0s	8,978	8,511	467	94.80%
5.0s	12,347	10,892	1,455	88.22%
6.0s	16,823	13,276	3,547	78.92%

The ETTC = 4.0s threshold exhibits superior performance characteristics that distinguish it as the optimal operating point. At this configuration, the system achieves the highest sensitivity (94.80%) among all tested thresholds, successfully capturing 8,511 of 8,978 identified conflicts as genuine near-miss events validated by observable evasive actions. This represents a 2.56 percentage point improvement over the 3.0s threshold (92.24%) and a 6.58 percentage point advantage over the 5.0s threshold (88.22%). Critically, the 4.0s threshold maintains this exceptional detection capability while preserving operational feasibility—the false positive count (467 events) remains substantially lower than higher thresholds (1,455 at 5.0s; 3,547 at 6.0s), avoiding the alert fatigue that would compromise real-world deployment.

The data reveals two fundamental limitations of alternative thresholds: Conservative thresholds (2.0–3.0s) achieve comparable sensitivity (92.24–92.25%) but identify only 34–68% of the conflict volume detected at 4.0s, suggesting insufficient temporal lead time to capture the full spectrum of safety-critical interactions in freeway diverging zones. Conversely, liberal thresholds (5.0–6.0s) dramatically expand detection scope but suffer from progressive sensitivity degradation (88.22% and 78.92%, respectively), indicating excessive contamination by routine traffic perturbations that do not manifest observable evasive behaviors—a clear marker of false positive proliferation.

The ETTC = 4.0s configuration therefore represents the inflection point where predictive lead time optimally aligns with behavioral validation criteria, maximizing true event capture while maintaining practical specificity for operational safety management systems.

#### 5.3.1 Risk factor analysis tool.

XGBoost is a black-box-like structure, and the influence of individual features within the model on decision-making and prediction generation is still not as intuitive as in linear models. Consequently, it cannot provide more specific guidance for control measures. To address this, this study employs a new model interpretation tool—SHAP (SHapley Additive exPlanation) [50], which can quantify the contribution of each feature to the model’s output.

Specifically, let the j-th feature of the *i*-th sample be *x*_*ij*_, the model’s prediction for *x*_*i*_ be *y*_*i*_, and the SHAP value of *x*_*ij*_ be *f*(*x*_*i*1_). Then the relationship between *y*_*i*_ and *f*(*x*_*i*1_) satisfies the following equation for an ensemble model of multiple trees:


$$y_{i}=y_{b}ase+f(x_{i}1)+f(x_{i}2)+\cdots +f(x_{i}k)$$ (26)

Where, *y*_*base*_ is the baseline of the entire model, typically equal to the mean prediction of all samples. From the equation, it can be seen that the SHAP value *f*(*x*_*ij*_) of the *j*-th feature represents that feature’s contribution to the prediction result. If *f*(*x*_*ij*_) is greater than 0, it indicates that the feature has a positive effect on the model, increasing the predicted value. Conversely, the feature has a negative effect, reducing the predicted value.

#### 5.3.2 Longitudinal conflicts.

**Fig 14:**
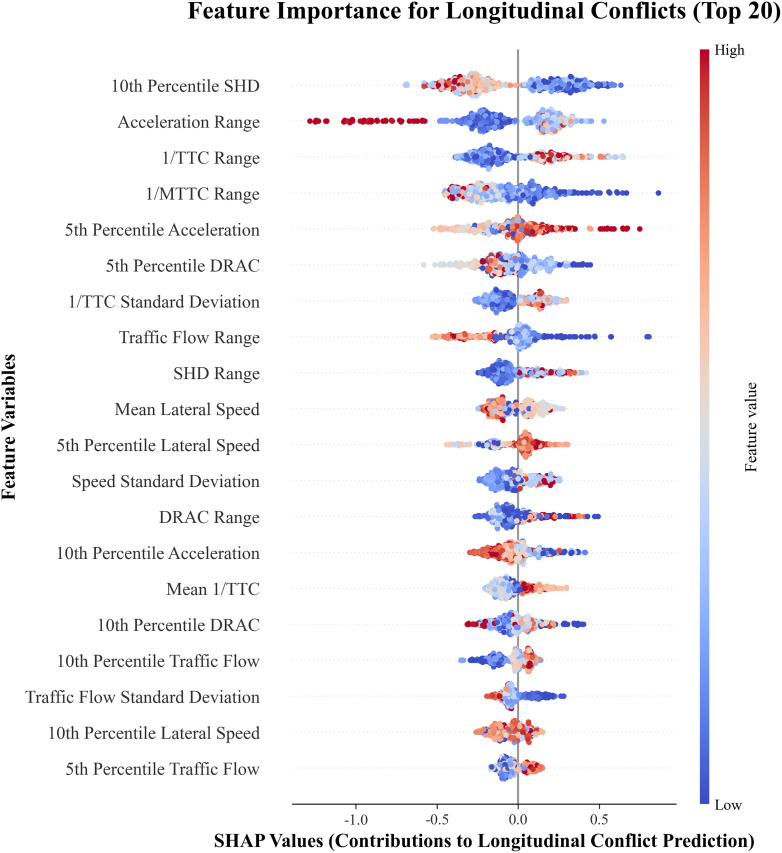
Key influencing factors of longitudinal crash risk.

Fig 14 presents the ranking of the top 20 features that have the greatest impact on the model’s prediction of a sample as a longitudinal conflict. The horizontal axis represents the SHAP values of each feature, with larger SHAP values indicating a greater contribution to predicting a conflict. Each point represents one sample, where redder colors indicate higher feature values and bluer colors indicate lower values.

For Six metrics most significantly impact longitudinal crash risk: the 10th Percentile SHD, Acceleration Range, 1/TTC Range, 1/MTTC, 5th Percentile Acceleration, and 5th Percentile DRAC. These metrics manifest as shorter stopping distances, larger acceleration fluctuations, greater inverse time-to-collision ranges, higher extreme deceleration values, and larger required deceleration rates, all indicative of elevated crash risk in interchange diverging areas.

The 10th Percentile SHD represents the stopping headway of vehicles with the shortest gaps. A smaller value indicates that at least 10% of vehicles in the traffic stream maintain an insufficient perception-braking distance. This deficit heightens their susceptibility to emergency braking and rear-end collisions when the lead vehicle decelerates or an unexpected event occurs [36].

A larger Acceleration Range indicates more intense longitudinal control fluctuations, characterized by frequent speed oscillations. This turbulent flow can generate acceleration/deceleration waves or even shockwaves. These waves amplify minor disturbances and propagate them along the traffic queue, consequently increasing the frequency of hazardous short-time headway situations [28].

A larger 1/TTC Range indicates greater variability in the intensity of vehicle approaches. This means brief periods of extreme proximity alternate frequently with periods of relaxed headways. Consequently, drivers must continuously make high-intensity longitudinal adjustments, which increases the probability of reaction delays or control overshooting, thereby elevating conflict risk [31].

A high 1/MTTC value indicates that the vehicle platoon sustained a low time headway, high-density car-following state throughout the observation period. This structural exposure drastically reduces the available reaction and braking distance in the event of a sudden disruption, thereby systematically increasing longitudinal crash risk [41].

The 5th Percentile Acceleration captures extreme deceleration events in the tail of the distribution. A larger (less negative) value signifies more frequent mandatory hard braking maneuvers. These events not only directly increase the probability of rear-end collisions but also generate backward-propagating deceleration waves, which degrade the safety margin for multiple following vehicles [28].

The 5th Percentile DRAC identifies situations where substantial braking effort is required even under moderately close-following conditions. This indicates a critically low systemic safety margin, necessitating high braking capability from drivers to avoid collisions, even when extreme proximity is not initially present [31].

**Fig 15:**
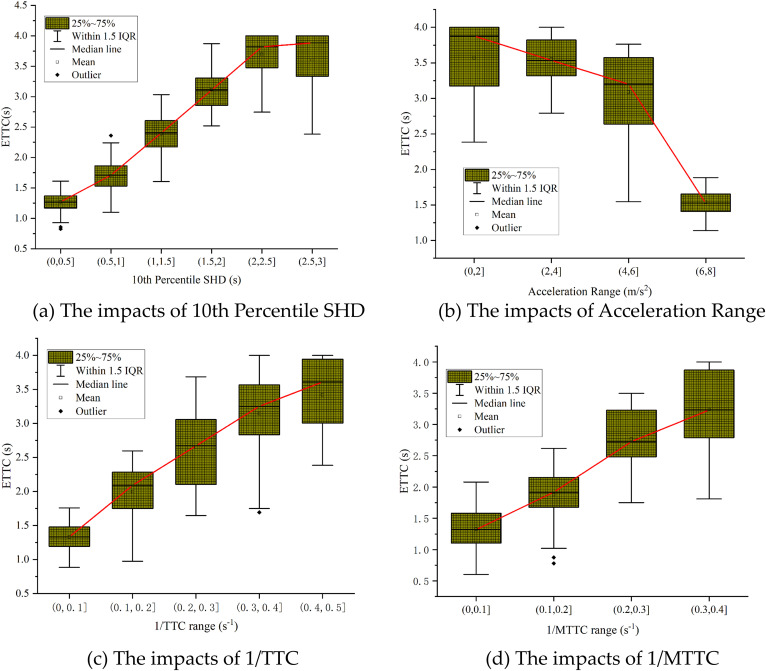
The impact of key Variables on Longitudinal Conflicts. **(a)** The Impacts of 10th Percentile SHD on longitudinal conflicts. **(b)** The Impacts of Acceleration Range on longitudinal conflicts. **(c)** The Impacts of 1/TTC range on longitudinal conflicts. **(d)** The Impacts of 1/MTTC range on longitudinal conflicts.

Since SHAP cannot quantify the association between variables and outcomes, a quantitative analysis was conducted on the top-ranked continuous variables to assess their impact on the longitudinal conflict risk (Fig 15).

(1) The 10th percentile SHD was segmented into 0.5s intervals, and the ETTC distribution of longitudinal conflicts within each interval was analyzed (Fig 15(a)). The severity of conflicts decreases with increasing SHD, and the ETTC distribution tends to concentrate. In the [0, 2]s interval, the severity of conflicts decreases sharply, while in the [2,3]s interval, the reduction in severity slows down, with most conflicts being general or minor. To ensure safe management in the diversion area, the roadside unit and the on-board unit should warn the driver when the headway is less than 2s, to encourage cautious lane changing [23].
(2) The range of segment acceleration was divided into intervals of 2 m/s², and the ETTC distribution of longitudinal conflicts within each interval was analyzed (Fig 15(b)). Longitudinal conflict risk increases significantly with increasing acceleration range, and the ETTC distribution tends to concentrate. The ETTC changes exhibit three stages: in the [0, 4] m/s² interval, the severity of conflicts increases slowly, with most being minor conflicts; in the [4,6] m/s² interval, the ETTC stabilizes at 3.07s, with most conflicts being minor or general; in the [6,8] m/s² interval, the severity of traffic conflicts increases rapidly, with most being severe conflicts.
(3) The 1/TTC range was segmented into 0.1 s^−1^ intervals, and the ETTC distribution of longitudinal conflicts within each interval was analyzed (Fig 15(c)). The ETTC generally increases with the increase in the 1/TTC range. In the [0, 0.1] s^−1^ interval, an increase in the 1/TTC range significantly increases the ETTC, and the severity of conflicts significantly decreases, with most conflicts in this interval being severe. In the [0.1, 0.4] s^−1^ interval, the increase in ETTC is smaller, with most conflicts being general. In the [0.4, 0.5] s^−1^ interval, the average ETTC value is the highest, with most conflicts being minor.
(4) The 1/MTTC range was segmented into 0.1 s^−1^ intervals, and the ETTC distribution within each interval was analyzed (Fig 15(d)). The ETTC value increases with the increase in 1/MTTC range, and the distribution range expands. In the [0.2, 0.4] s^−1^ interval, most conflicts are minor or general, with fewer severe conflicts; whereas severe conflicts are concentrated in the [0, 0.1] s^−1^ interval.

#### 5.3.3 Lateral conflicts.

**Fig 16:**
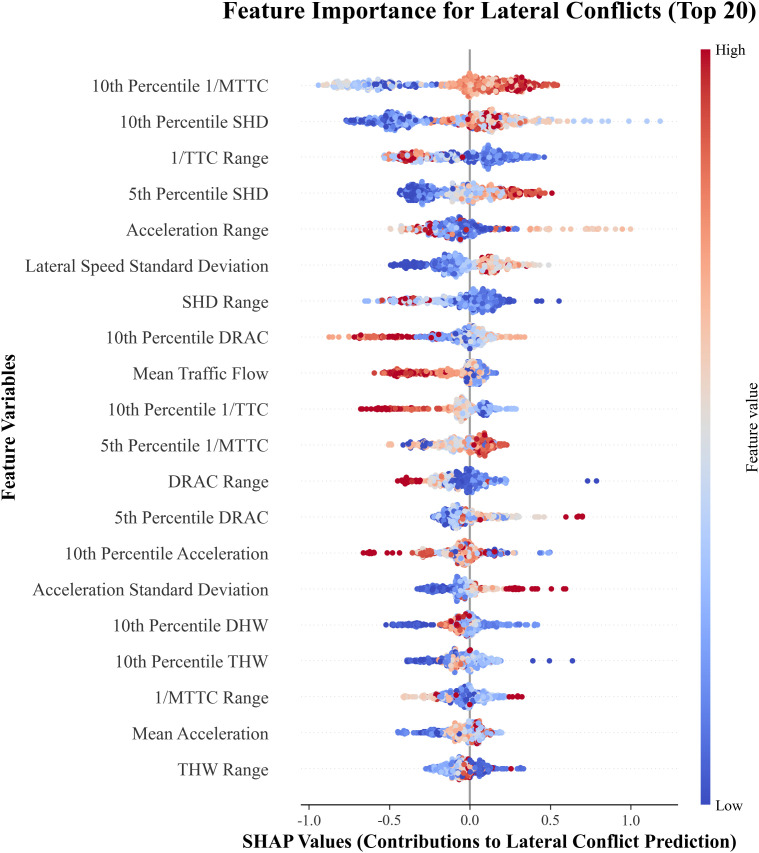
Key influencing factors of lateral crash risk.

Fig 16 ranks the 20 most important features influencing lateral crash risk severity. The top four influencing factors are all Surrogate Safety Measures (SSMs), confirming their significant effectiveness in capturing segment-level lateral crash risks. Among these, the 10th Percentile Modified Time to Collision (1/MTTC) exerts the most substantial influence on the model’s predictions. A higher 1/MTTC value corresponds to an increased lateral crash risk. This relationship likely arises because a shorter modified time to collision indicates insufficient lateral spacing, forcing drivers to execute emergency maneuvers to avoid accidents [31].

Furthermore, other critical SSMs—including the 10th Percentile Stopping Headway Distance (SHD), Inverse Time to Collision (1/TTC), and 5th Percentile SHD—also contribute positively to the model. This indicates that shorter stopping distances and greater variability in conflict proximity metrics effectively capture complex vehicular interactions, thereby enhancing the model’s lateral risk identification capability.

Notably, unlike for longitudinal conflicts, macroscopic features like Mean Traffic Flow and Mean Acceleration are more significant predictors of lateral crash risk. Elevated traffic flow and acceleration increase lateral crash risk. This increase occurs primarily because high traffic flow induces more frequent lane-changing maneuvers, while greater acceleration can exacerbate vehicle instability. Features describing volatility, specifically the standard deviation of lateral speed and the acceleration range, also exhibit high importance. This reflects the significant impact of fluctuations in lateral vehicle control on crash risk.

**Fig 17:**
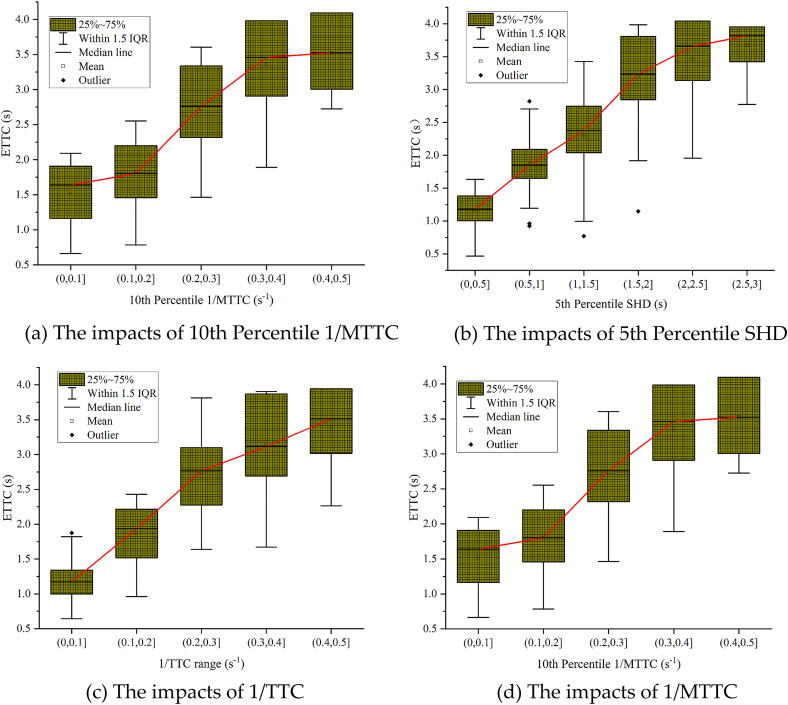
The impact of key variables on longitudinal conflicts.

Similarly, a quantitative analysis of the top-ranked key influencing factors for lateral crash risk was conducted to evaluate their impact on longitudinal conflict risk (see Fig 17).

(1) The 10th Percentile 1/MTTC was segmented into 0.1 s intervals, and the ETTC distribution of lateral conflicts within each interval was analyzed (Fig 17(a)). Conflict severity decreases as the 10th Percentile 1/MTTC increases. Within the [0, 0.2] s interval, lateral conflict severity declines slowly, with ETTC values predominantly below 2 s; whereas in the [0.2, 0.3] s interval, the severity reduction rate increases abruptly, and conflicts in this range are primarily moderate conflicts; within the [0.3, 0.5] s interval, the severity reduction rate slows again, and conflicts are mostly minor conflicts. To ensure safety management in diverge areas, the 10th Percentile 1/MTTC range of 2.5–3 s may serve as a threshold for proactive safety control [27].
(2) The 5th Percentile SHD was segmented into 0.1 s intervals, and the ETTC distribution of longitudinal conflicts within each interval was analyzed (Fig 17(b)). Longitudinal conflict risk increases significantly with larger acceleration ranges, and the ETTC distribution becomes more concentrated. ETTC variation exhibits three phases: within [0, 4] m/s², conflict severity increases slowly and conflicts are mostly minor; within [4, 8] m/s², ETTC stabilizes at 3.07 s with primarily minor and moderate conflicts; within [8, 12] m/s², traffic conflict severity rises rapidly and conflicts are predominantly severe.
(3) The 1/TTC range was segmented into 1 s^−1^ intervals, and the ETTC distribution of longitudinal conflicts within each interval was analyzed (Fig 17(c)). ETTC exhibits an overall increasing trend with higher 1/TTC range values, indicating significantly reduced lateral crash risk. Within the [0, 0.3] s^−1^ interval, lateral crash risk decreases sharply, and conflict events shift from severe to moderate conflicts; whereas in the [0.3, 0.5] s^−1^ interval, the average ETTC value rises at a decelerated rate, with conflicts predominantly being moderate and minor types.
(4) The 10th Percentile 1/MTTC was segmented into 0.1 s^−1^ intervals, and the ETTC distribution within each interval was analyzed (Fig 17(d)). ETTC values increase with higher 10th Percentile 1/MTTC values. Within the [0, 0.2] s^−1^ interval, the ETTC growth trend is relatively slow, with all values below 2.75 s; in the [0.2, 0.4] s^−1^ interval, the ETTC growth rate accelerates significantly, and lateral conflicts are predominantly moderate conflicts; whereas ETTC values in the [0.4, 0.5] s^−1^ interval show minimal distributional differences from the [0.3, 0.4] s^−1^ interval, with both intervals concentrated in minor conflicts.

#### 5.3.4 Summary of key factors influencing crash risk.

The different sets of dominant predictors for longitudinal and lateral conflicts revealed by the SHAP analysis are consistent with the inherent distinction in their formation mechanisms. Longitudinal conflicts in interchange diverging areas are essentially car-following events along the traffic stream. Their escalation is governed by how individual drivers regulate spacing and speed relative to the leading vehicle; thus, percentile-based SSMs such as SHD, TTC/MTTC ranges, and acceleration range—which summarize extreme short-headway states and longitudinal control instability—naturally become the most influential predictors of longitudinal crash risk. In this sense, longitudinal conflicts are predominantly driven by microscopic vehicle control behaviors within existing lane configurations.

In contrast, lateral conflicts are intrinsically linked to lane-changing maneuvers required for vehicles to reach the exit ramp. The decision to change lanes, the availability of acceptable gaps in both the current and target lanes, and the interaction between diverging and through traffic all depend strongly on the prevailing macroscopic traffic state. When mainline and ramp traffic volumes are high, vehicle density increases and usable gaps shrink, forcing drivers to accept shorter time gaps and execute more aggressive lateral maneuvers. This mechanism explains why macroscopic indicators such as Mean Traffic Flow and Mean Acceleration, together with SSMs reflecting minimum lateral safety margins (e.g., 10th percentile 1/MTTC, 5th percentile SHD), play a more prominent role in lateral conflict prediction than in longitudinal conflict prediction in our model.

Similar patterns have been reported in previous empirical studies. Ma et al. found that in expressway diverging areas, conflict risk increased with mainline and ramp traffic volumes, while longer deceleration lanes reduced conflicts, highlighting the influence of macroscopic flow and geometry on diverging-area safety. Zhao et al. [27] showed that under congested traffic states, severe conflict occurrence is more sensitive to changes in traffic volume and ramp proximity, whereas under near free-flow conditions, severe conflicts are mainly triggered by unsafe microscopic behaviors such as high speeds and short spacings [41]. Zhang et al. [50] further demonstrated that, in expressway diverging areas, the interaction between high lane-change frequency and speed fluctuation is the dominant driver of conflict risk, again confirming that lane-changing risks are co-determined by microscopic driving behavior and macroscopic flow conditions. Our findings therefore reinforce the view that longitudinal and lateral conflicts reflect two distinct but coupled risk formation mechanisms, and that effective proactive safety management must account for both microscopic control instability and macroscopic traffic-flow states.

In summary, the feature SHAP values demonstrate that the surrogate safety measures (SSMs) introduced in our road segment crash risk prediction model are significant contributors, particularly for predicting longitudinal risk. The influence of these aggregated SSMs on segment-level risk is consistent with their expression of risk at the individual vehicle level. Consequently, unsafe vehicle interactions emerge within a road segment prior to a conflict. As more vehicles exhibit risky behaviors, SSM features characterizing conflicts become more prevalent. Thus, micro-level individual risks gradually aggregate into segment-level risks. Among the aggregated metrics, the 5th and 10th percentile values of SSMs provide a more sensitive characterization of segment-level crash risk than the mean value. These low quantiles approximate the “worst-case” safety margins experienced by the most exposed 5–10% of vehicles within each 30-second interval, such as the shortest stopping headways or the highest required decelerations. When traffic conditions deterio-rate and more vehicles enter critical states, the lower tail of the SSM distribution shifts rapidly, and the 5th/10th percentile values decrease (or increase, depending on the indicator’s definition) accordingly. By contrast, the mean SSM remains buffered by the large number of vehicles that still operate under relatively safe conditions, and thus is less responsive to the emergence of high-risk interactions. Consequently, the SHAP analysis assigns markedly higher importance to percentile-based SSM features than to their mean counterparts, confirming that crash risk in diverging areas is driven by the accumulation of a small fraction of highly risky interactions rather than by average traffic conditions. This risk aggregation process is particularly pronounced prior to lateral conflicts. For these events, nearly all critical predictive features are SSMs. Furthermore, these SSMs encompass temporal, spatial, and deceleration-related metrics, indicating that a diversity of SSM types enhances model robustness more effectively than any single metric type.

Beyond SSMs, speed and traffic flow parameters—the most frequently used variables in existing research—also contribute significantly. The influence of traffic flow parameters on conflict prediction aligns with findings from Xu et al. [10] and Yang et al. [14]. Both sharp acceleration changes and an increased mean acceleration elevate crash risk. Furthermore, traffic flow metrics (e.g., mean traffic flow and traffic flow range) substantially influence both conflict types. High flow volumes and fluctuations increase the probability of segment-level crashes. These parameters are critical for both conflict types and are key determinants of traffic conflict risk.

## 6 Conclusions

Utilizing vehicle trajectory data extracted by drones from multiple expressway diverging zones, this study proposes an ETTC-based traffic conflict assessment metric incorporating two-dimensional vehicle interaction characteristics, conducting in-depth analysis of conflict types, spatiotemporal distribution patterns, and severity levels. Results indicate ETTC thresholds of 1.38 s (slight/moderate) and 3.64 s (severe/moderate) for longitudinal conflicts, while lateral conflict thresholds are 1.41 s (slight/moderate) and 3.64 s (severe/moderate). Using XGBoost modeling and SHAP interpretability framework, key factors influencing longitudinal and lateral conflicts in diverging zones were identified: longitudinal conflicts are primarily driven by stopping sight distance (10th percentile SHD), acceleration range reflecting driving instability, and time-criticality fluctuation (1/TTC range); lateral conflicts are predominantly influenced by modified collision time urgency (10th percentile 1/MTTC), minimum safety margin (5th percentile SHD), and lane-change trajectory disorder characterized by lateral acceleration standard deviation. Furthermore, SSM percentile metrics demonstrated significantly higher sensitivity in risk characterization than their mean value counterparts, confirming the dynamic process where micro-level risk accumulation evolves into segment-level collective risk, providing novel theoretical interpretation for conflict formation mechanisms. This study’s findings offer two pivotal implications for engineering applications: First, real-time control systems can dynamically deploy lane control strategies and speed guidance measures based on a 60-second warning window. Specifically, integrating multi-source sensors (e.g., roadside cameras, radar) with AI enables automatic identification of traffic conflict types and ETTC metrics; for persistent severe conflicts, the system issues warnings via variable message signs (VMS), roadside broadcasts, or in-vehicle units to prompt risk-averse driving maneuvers.
